# SM-SegNet: A Lightweight Squeeze M-SegNet for Tissue Segmentation in Brain MRI Scans

**DOI:** 10.3390/s22145148

**Published:** 2022-07-08

**Authors:** Nagaraj Yamanakkanavar, Jae Young Choi, Bumshik Lee

**Affiliations:** 1 Department of Electronics and Communications Engineering, CHRIST University, Bangalore 560029, India; nagaraj.yamanakkanavar@christuniversity.in; 2 Division of Computer Engineering, Hankuk University of Foreign Studies, Yongin 17035, Korea; jychoi@hufs.ac.kr; 3 Department of Information and Communications Engineering, Chosun University, Gwangju 61452, Korea

**Keywords:** brain MRI, combined-connection, convolutional neural network, fire module, tissue segmentation

## Abstract

In this paper, we propose a novel squeeze M-SegNet (SM-SegNet) architecture featuring a fire module to perform accurate as well as fast segmentation of the brain on magnetic resonance imaging (MRI) scans. The proposed model utilizes uniform input patches, combined-connections, long skip connections, and squeeze–expand convolutional layers from the fire module to segment brain MRI data. The proposed SM-SegNet architecture involves a multi-scale deep network on the encoder side and deep supervision on the decoder side, which uses combined-connections (skip connections and pooling indices) from the encoder to the decoder layer. The multi-scale side input layers support the deep network layers’ extraction of discriminative feature information, and the decoder side provides deep supervision to reduce the gradient problem. By using combined-connections, extracted features can be transferred from the encoder to the decoder resulting in recovering spatial information, which makes the model converge faster. Long skip connections were used to stabilize the gradient updates in the network. Owing to the adoption of the fire module, the proposed model was significantly faster to train and offered a more efficient memory usage with 83% fewer parameters than previously developed methods, owing to the adoption of the fire module. The proposed method was evaluated using the open-access series of imaging studies (OASIS) and the internet brain segmentation registry (IBSR) datasets. The experimental results demonstrate that the proposed SM-SegNet architecture achieves segmentation accuracies of 95% for cerebrospinal fluid, 95% for gray matter, and 96% for white matter, which outperforms the existing methods in both subjective and objective metrics in brain MRI segmentation.

## 1. Introduction

The segmentation of the brain on magnetic resonance imaging (MRI) automatically provides a quantitative assessment of pathologies and is useful for monitoring disease progression. Over the last few decades, MRI has made remarkable progress in evaluating brain injuries and investigating brain anatomy. The MRI can detect disorders related to the brain, such as Alzheimer’s disease (AD) where the diagnoses of the diseases are commonly made through tissue segmentation. Brain MRI segmentation at different times is also utilized to measure structural changes in the brain. An accurate assessment of disorders such as AD depends equally on detecting and identifying diseased tissue and its surrounding healthy structures. Thus, MRI is a prevailing modality for the analysis of brain tissues, including white matter (WM), cerebrospinal fluid (CSF), and gray matter (GM) [1]. Furthermore, in comparison to other modalities (e.g., positron emission tomography and computed tomography), MRI provides superior spatial contrast images with higher spatial resolutions without having any harmful effects on human health [2]. Over the past two decades, MRI imaging has made tremendous advances in detecting brain injuries and examining their anatomy [3]. Brain-tissue segmentation is a challenging task due to defects in the neurological anatomy of the brain. These brain tissues are essential components in computer-aided diagnosis and neuroscience research, and they assist in the detection of different diseases. The purpose of segmenting brain tissue is to facilitate effective analysis of the volumetric developments (i.e., surface area, gyrification, and cortical thickness) of GM and WM; this offers significant indications about the initial progress of neuroanatomical diseases. Owing to the complicated structure of the brain and the irregular boundaries between different tissues, brain MRI segmentation is a difficult task [4]. To segment the object in an accurate and computationally efficient way, numerous machine learning approaches have been introduced, based on clustering algorithms [5,6], level set methods [7,8], and pattern recognition approaches [9,10]. However, the performance of these approaches is often hindered by brain structure complexity, low soft-tissue contrasts, non-uniform intensity, the partial volume effect, and MRI noise [11]. In addition, feature extraction does not produce accurate segmentation results of the brain MRI in the presence of image noise and other imaging artifacts. In this case, more powerful and discriminatory features are needed to integrate spatial interaction across intensities.

In medical images, deep learning techniques have recently been applied to provide accurate and efficient tissue segmentation [12]. Considerable research efforts based on deep learning approaches have been successfully made to solve problems for denoising of MRI, motion artifacts in MRI due to the relatively long acquisition time, and suffering from a low signal-to-noise ratio [13], which are problems inherent to MRI and not easy to be solved by the classical machine learning approaches because they strongly depend on the quality of data and are highly susceptible to errors which lead to inability to interpret the data accurately. In light of this fact, Coupe et al. [14] used a two-stage deep learning technique for noise reduction on MRI. The initial step is to use a convolutional neural network (CNN) to remove noise from the images without estimating the local noise level. The filtered image is then employed as a guide image in a non-local means filter that is rotationally invariant. This method shows promising results for denoising of MRI scans. A common problem with the MRI is that it often suffers from a low signal-to-noise ratio, such as diffusion-weighted imaging (DWI) and 3D MRI scans. To cope with this problem, Jiang et al. [15] proposed the multi-channel feed-forward noise removal CNNs while Ran et al. [16] introduced residual encoder–decoder Wasserstein GANs to reconstruct noise-free 3D MRI images. The MRI is also susceptible to image artifacts because of the very long acquisition time. Kustner et al. [17] introduced a non-reference method for automatically detecting the appearance of motion artifacts on MRI. The motion artifacts were assessed on a per-patch basis using a CNN classifier, then utilized to localize and estimate the motion artifacts on a test data set.

Since a deep learning technique does not require manual steps to learn important representations and features, the amount of time for manual steps in traditional machine learning techniques can be reduced. As described in [14,15,16,17], deep learning methods are characterized by the focus on feature learning, which is learned automatically by analyzing the data. This results in higher performance than traditional methods. In particular, the developed deep convolutional neural networks (DCNNs) have shown higher performances in numerous medical segmentation tasks [18]. The DCNN has a better-characterized capability compared to traditional methods and can automatically identify the most valuable information from large datasets. The primary disadvantage of existing DCNN-based approaches is that during pooling for feature extraction, they can lose spatial information, which might reduce the model accuracy, especially when dealing with a variety of shapes and object positions.

To overcome this problem, several deep networks for segmentation (e.g., SegNet [19], U-net [20], and M-net [21] architectures) have been developed. The SegNet architecture with an encoder–decoder architecture was originally developed by Badrinarayanan et al. [19] for the autonomous-driving car system problem. However, it shows a crucial drawback because neighboring information is often lost while pooling low-resolution feature maps. Ronneberger et al. [20] introduced a U-net architecture for medical image segmentation and used a skip-connection strategy to concatenate feature maps from the encoder to the decoder blocks. However, U-net applies many learnable parameters during the training stage, increasing its computation time compared to other models. Adiga et al. [21] used the M-net architecture for fingerprint image noise removal and inpainting; however, even though feature channels increase over the input images during the down- and upsampling phases, the model required a lot of memory to store large numbers of parameters for high-resolution input images. Moreover, when entire images are used as network inputs, the model is at risk of losing local information. These conventional methods [22,23] were designed to include cropping and convolution operations between up-convolution and down-convolution parts to improve segmentation accuracy. In the fully convolutional networks (FCN) architecture, short skip connections were added similar to those found in residual networks, where input blocks are skipped through their outputs to construct very deep CNNs [23]. The approach in [24] combines upsampled feature maps with those skipped along the contractive path, while in [20], the combined features are concatenated, and nonlinearity is added between each upsampling stage. All these existing skip connections implement features copied from the contracting network layer to the corresponding upsampling layer. Furthermore, the derivatives of network backpropagation are used to assess deep neural network gradients because they are transferred layer-wise from the final to the initial layer. To estimate the derivatives of each layer, the final layer derivative is multiplied by the initial layer derivative. In general, the weights and biases of initial layers cannot be effectively updated until the gradient gets smaller with each training session. The small gradients reduce overall network accuracy because these initial layers are often important for recognizing the crucial components of the input data [25].

To overcome the aforementioned limitations, we propose the so-called “lightweight SM-SegNet architecture” for automatic brain tissue segmentation on MRI. The proposed architecture includes general M-Net features such as multi-scale inputs (left leg) and deep supervision (right leg); in particular, it involves novel long skip connections, fire modules, and combined-connections (including both the skip connection and pooling indices). The downsampled input in the left-leg path extracts discriminative information and improves feature representations at the encoder layer. The right-leg path with upsampled decoder layer output can make the model convergence faster and address the vanishing gradient problem [26]. As a result, the proposed network extracts discriminative information from both side layers; thus, reducing the gradient problem and improving the overall derivative of the network layer. In addition, the combined-connections pass features from the encoder to the decoder path to recover the spatial information (absent during down-sampling) and lead to faster convergence of the model. The proposed long skip connections can reuse the network features and stabilize training and convergence, significantly improving segmentation accuracy. The fire modules (with the squeeze and expand layers) produce significantly fewer parameters with efficient memory utilization. The proposed model was then trained from the entire input slice of the MRI scan using uniform patches of the same size. As proven in our earlier study [27,28], the partition of uniform patches facilitates brain MRI localization by focusing on fine details within each patch. Based on multiple evaluation metrics, the proposed approach demonstrates significant improvements over recently developed brain MRI segmentation methods. The following is the list of our major contributions:

- We propose “lightweight SM-SegNet,” a fully automatic brain tissue segmentation on MRI, using a multi-scale deep network integrated with a fire module.
- Our SM-SegNet architecture represents an end-to-end training network that applies an M-shape convolutional network with multi-scale side layers at the input side to learn discriminative information; the upsampling layer at the output side provides deep supervision.
- The proposed long skip connections stabilize the gradient updates in the proposed architecture, improving the optimization convergence speed.
- The encoder and decoder (designed with fire modules) reduce the number of parameters and the computational complexity, resulting in a more efficient network for brain MRI segmentation.
- We propose using a uniform division of patches from brain MRI scans to enhance local details in the trained model; this minimizes the loss of semantic features.

This paper is organized as follows: Section 2 focuses on related studies. In Section 3, we describe in detail the proposed method and its architecture. Section 4 provides experimental conditions, comparisons, and comprehensive analyses of the proposed method. Finally, our conclusions are presented in Section 5.

## 2. Materials and Methods

Automatic brain tissue segmentation on MRI not only provides a strong basis for pathological evaluation but also helps medical doctors accurately diagnose diseases. Precise automated segmentation of brain tissues (e.g., GM, WM, and CSF) in MRI is of great importance for the quantitative study of brain tissue and large-scale intracranial volumes. At present, researchers primarily use semantic- and patch-wise strategies for brain MRI segmentation. Milletari et al. [29] proposed a segmentation method using the Hough convolutional neural network (CNN), which is based on the Hough vote, a technique for automatically localizing and segmenting anatomies of interest. Zhenglun et al. [30] proposed the tissue images segmentation method, where a multi-scale wavelet transformation was utilized for pre-processing, then the MRI brain was segmented using a CNN. Ren et al. [31] introduced adversarial defense and task reorganization for brain MRI segmentation on a limited number of datasets. The training data was augmented using adversarial defense, and task reorganization was used to incorporate higher-level features to the pixel-level segmentation task. A 3D CNN for the automatic segmentation of neuroanatomies from T1-weighted MRI was proposed in [32], where the network learned an abstract feature representation and performed multiclass classification in brain MRI. Jie et al. [33] presented DCNN-based segmentation strategies for brain MRI segmentation using different input images of variable sizes and views. A convolutional autoencoder was used to reconstruct images based on the probabilistic atlas of brain anatomy. Zhou et al. [34] proposed the U-net++ architecture, which has encoder and decoder blocks coupled with a number of layers and dense skip routes for medical image segmentation. A drawback in U-net++ is to significantly increase the number of parameters by using dense connections [35]. Gu et al. [36] proposed the CE-Net, a context encoder network (CE-Net) that leverages a pre-trained ResNet block within the encoder to aid the segmentation of medical images. Kong et al. [37] used discriminative clustering and a feature selection approach to investigate the segmentation of brain tissues on MRI. Deng et al. [38] propose an FDNN that derives information simultaneously from neural and fuzzy representations. In [39], an M-SegNet architecture with global attention was proposed for brain MRI segmentation as in our previous study, where the global attention approach captures rich contextual information by combining local features and global dependencies at the decoding stage.

According to recent research, most deep neural networks techniques tend to be over-parametrized, causing network redundancies as well as excessive memory and processing resource consumption. To reduce redundancy and shrink models in these huge parameter spaces, various compression approaches (e.g., downsizing, factorizing, or compressing pre-trained networks) are used [40]. Singular value decomposition is commonly applied to a pre-trained CNN architecture in the model-compression method to provide lower-order parameter estimations. In the network pruning approach, the parameters of the pre-trained model lower than a given threshold are replaced with zeros to produce sparse matrices, resulting in the fewer bits of indices by using relative index encoding. To reduce the computational complexity, different convolution-kernel-factorization-based techniques are followed. Depthwise separable convolution is implemented in SqueezeNet [41], which is a convolution factorizing method that splits convolutions over channels rather than within them. The model size can be decreased by quantization, which reduces the data’s dynamic range from 32 to 8 or 16 bits. Existing methods mostly focus on how to design efficient network computation to reduce the number of model parameters and the inference time. The methods have been used to develop many classic lightweight CNN models, such as MobileNet [42], ThunderNet [43], ShuffleNet [44], and SqueezeNet [41]. The MobileNet [42] shows reduced parameters and performs faster, but the results are less accurate than other state-of-the-art networks. Although reducing the number of parameters would have several benefits, such as a smaller network and faster model training, this is not a simple task because reducing the model capacity can also lead to a loss of accuracy. Thus, a delicate balance has to be maintained between complexity and performance.

Our proposed method is partially related to the following previous works in that there are several applications related to squeeze and excitation (SE) networks for the segmentation of medical images. Roy et al. [45] proposed three variants of the SE modules for semantic segmentation and learning attention at the channel and spatial levels, although all feature maps are derived from a single spatial attention map. Pereira et al. [46] modified the U-Net with innovative features of recombination and calibration and separated sub regions of the tumor into a hierarchy to take advantage of its hierarchical structure. Oktay et al. [47] introduced an attention U-Net that can detect fine features in medical images, best suited for lesion segmentation. Qin et al. [48] proposed the autofocus convolutional layer to improve the effectiveness of neural networks adopting multi-scale processing. After merging multi-modal images in the input space, a convolutional layer is utilized to modify the size of the receptive field with different dilation rates. The fire module is a lightweight structure with fewer parameters to learn and requires less computation complexity.

**Figure 1:**
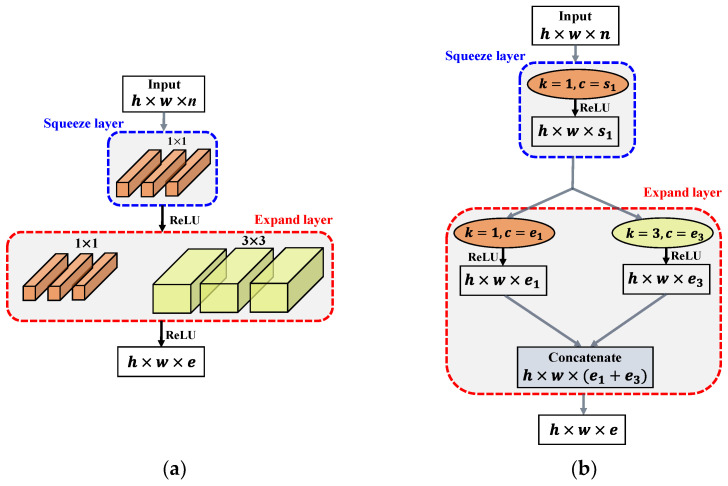
Schematic representation of the fire module. (**a**) Fire module structure and (**b**) workflow of the fire module.

Furthermore, the fire module has 1 × 1 and 3 × 3 convolution kernels, which not only achieve the optimal classification accuracy but also decomplexify the network [49]. Figure 1a shows the fire module structure, which includes squeeze and expand layers. The squeeze layer uses a 1× 1 convolutional kernel to reduce the input elements and minimize the number of input-element channels. For multi-scale learning, the expansion layer employs 1× 1 and 3 × 3 convolutional kernels. Figure 1b shows the workflow process of the fire module. The input size of the feature map is h×w×n (height × width × channel). First, the input feature maps that were fed into the squeeze layer, to generate the $h\times w\times s_{1}$ output feature maps. The sizes of the feature maps are not modified, though the number of channels decreases from *n* to $s_{1}$. The output feature maps of the squeeze layer are fed into 1 × 1 and 3 × 3 convolutional kernels in the expanded layer. The $e_{1}$ and $e_{3}$ are the number of input filters with 1 × 1 and 3 × 3 kernels, respectively. In order to make the output activation of the filters of the expand layer (with the size of 1 × 1 and 3 × 3 pixels) have the same dimension, the boundary zero-padding operation is performed with 3 × 3 filters as input in the expand layer. The fire-module output is formed by concatenating the two parallel convolution output channels [50]. These two group convolutions fuse features to improve the predictive segmentation accuracy for brain MRI scans. Furthermore, the number of channels after concatenation is changed to $e_{1}+e_{3}$. Finally, the expansion and squeeze layers are activated by a rectified linear unit (ReLU). Thus, the fire module helps to maintain a competitive accuracy with minimal learnable parameters.

## 3. Proposed Methodology

As discussed in Section 1, SegNet, U-net, and M-net suffer severe shortcomings, including the loss of neighbor or local information [28,51] and computational burden, despite their promising segmentation performances. To resolve these problems and enhance the performance of the segmentation procedure, we propose a novel SM-SegNet architecture that utilizes side paths, combined-connections, long skip connections, and uniform patches. The side paths extract the multi-scale information and implement combined-connections from the encoder to the decoder to recover spatial information. Furthermore, long skip connections stabilize the gradient updates in the network, and the uniform input patches highlight the local details in the input image. In particular, we adopt a fire module to reduce both the memory demands and computational complexity of the proposed architecture. There are two subsections in the proposed model: (i) the outline and (ii) the proposed SM-SegNet architecture.

### 3.1. Outline of the Proposed Method

**Figure 2:**
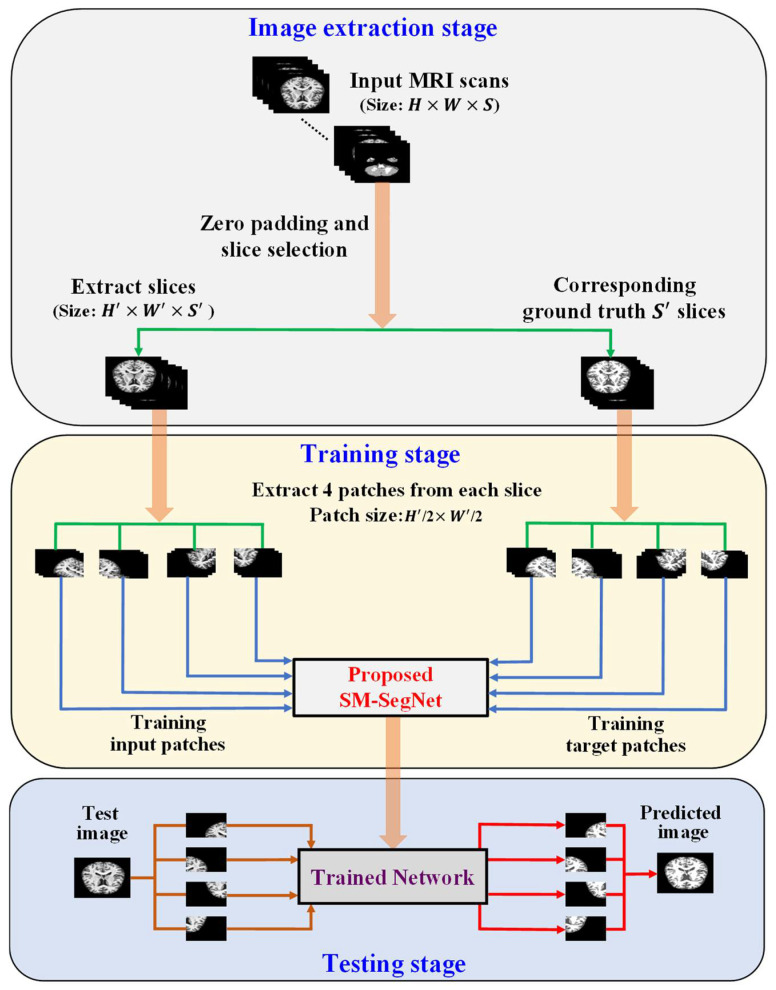
The pipeline for the overall process of the proposed method.

Figure 2 shows the overall procedure of the proposed architecture. In general, each MRI scan has dimensions of height × width × slices (*H* × *W* × *S).* As shown in Figure 2, we create uniform patch sizes $H^{\prime }\times W^{\prime }$ by padding zeros throughout the boundaries of the image. In our previous work [39,52], several slices at the beginning and end of the brain MRI volume contain no useful information, and almost identical information will be exchanged between consecutive slices. As a result, to eliminate non-informative slices, $S^{\prime }$ slices are extracted from the S slices in a constant interval of 3. To train the proposed SM-SegNet architecture, the extracted slices of each MRI scanning and their respective ground truths are divided into uniform patches of uniform dimensions of $H^{\prime }/2\times W^{\prime }/2$ and fed into the network.

The SM-SegNet architecture is composed of left- and right-leg paths, long skip connections, combined-connections, and fire modules. The left-leg paths downsample inputs using maximum pooling layers to extract discriminative information that feeds the corresponding encoder layer. Similarly, for the right-leg path, the outputs of the decoding layer are also upsampled to the size of the input. Furthermore, the decoder layer and the right leg results in a combined output that accelerates convergence and addresses the vanishing gradient problem. Thus, the combination of left and right legs increases the effectiveness of network training. The combined-connections are used to restore spatial information missed during down-sampling and improve convergence by transferring features from the encoder to the decoder blocks. In addition, long skip connections are used to stabilize the gradient updates in the network

### 3.2. Outline of the Proposed Method

**Figure 3:**
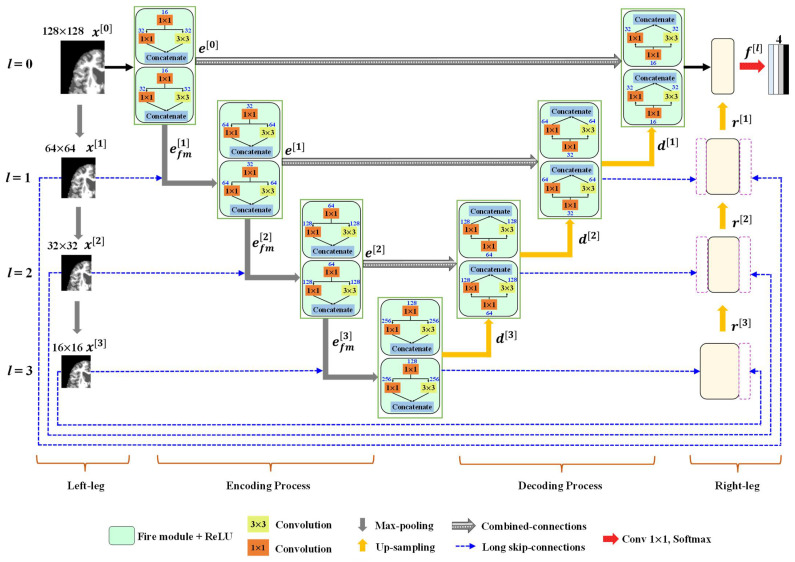
The overall architecture of the proposed SM-SegNet model. The fire modules are shown as solid boxes, with the set of feature maps indicated on the top of each box.

Figure 3 illustrates the overall architecture of the proposed SM-SegNet model featuring a fire module. As shown in Figure 3, the proposed network architecture is an end-to-end learning structure that comprises multi-scale input and deep supervision, novel long skip connections, combined-connections, and fire modules. The encoder and decoder block mechanisms are described in detail as follows:

#### 3.2.1. Encoder Block

In our proposed architecture, every layer in the encoder path consists of two fire modules with convolutional kernel sizes of 1 × 1 and 3 × 3 for multi-scale learning. The fire modules are feedforward networks that map the output of the *l*-th layer to an input to the (*l* + 1)-th layer, as

(1) $$s_{fm}^{\left[l\right]}=H\left(x^{\left[l\right]}*w_{1\times 1}^{\left[l\right]}+b^{\left[l\right]}\right)$$

(2) $$e_{fm}^{\left[l\right]}=concatenate\left(H\left(s_{fm}^{\left[l\right]}*w_{1\times 1}^{\left[l\right]}+b^{\left[l\right]}\right),\;\;\;\;H\left(s_{fm}^{\left[l\right]}*w_{3\times 3}^{\left[l\right]}+b^{\left[l\right]}\right)\right)$$

where $x^{\left[l\right]}$ is the input sample; $s_{fm}^{\left[l\right]}$ is the squeeze layer output of the fire module; $e_{fm}^{\left[l\right]}$ is the expanded layer output of the *l*-th fire module; $w_{1\times 1}^{\left[l\right]}$ and $w_{3\times 3}^{\left[l\right]}$ are the kernel weights, where subscripts 1 × 1 and 3 × 3 refer the size of the kernels; $b^{\left[l\right]}$ is the bias parameter; ∗ is the convolution operator; and $H\left(\cdot \right)$ is the ReLU activation function. Using a 2 × 2 max-pooling operation with a stride of 2, the output of the fire module is down-sampled. The max-pooling method minimizes image dimensions while preserving fine feature map features. The input image is down-sampled by 2 × 2 max-pooling with a stride of 2 in the left leg of the architecture and then appended to the corresponding encoder layer using side-skip connections. The deep layers use these multi-scale inputs to help to extract discriminative information. On the encoder side, the *l*-th layer produces the overall feature maps as

(3) $$e^{\left[l\right]}=concatenate\left[pool\left(e_{fm}^{\left[l\right]},2\right),pool\left(x^{\left[l\right]},2\right)\right]$$

where $e^{\left[l\right]}$ represents the final output of each encoder layer. The fire module features are passed from the encoder layer to the corresponding decoding layer using combined-connections. The combined-connections are applied in our proposed method, which is based on the skip connections and the pooling of indices from the encoder to the decoder path, in order to restore spatial features that have been lost due to downsampling and improve convergence. The combined-connections in the proposed architecture are highlighted by the blue and gray arrows in Figure 3.

#### 3.2.2. Decoder Block

Each decoding layer comprises two consecutive fire modules. The max-pooling operation at the decoder side is replaced by un-pooling layers [19], which upsample the feature maps instead of using learnable parameters. The pooling layer uses the pooling indices to upsample the input feature maps with spatial dimensions during max-pooling of the corresponding encoder block. The aforementioned un-pooled feature maps are concatenated via skip connections to encoder feature maps with similar spatial dimensions. These skip connections transfer the features from the encoder to the decoder so that spatial information can be well recovered; thus, resulting in faster convergence of the model. The output of each decoder layer with a combined-connection is expressed as

(4) $$d^{\left[l\right]}=concatenate\left[\left(unpool\left(x^{\left[l-1\right]},p_{ind\;}^{\left[l\right]}\right)\;,e^{\left[l\right]}\right)\right]$$

where $d^{\left[l\right]}$ is the output of the decoder layer, $p_{ind\;}^{\left[l\right]}$ represents the pooling indices taken from the encoder layer and inputted to the decoder layer for faster training, and $e^{\left[l\right]}$ represents the encoder layer features passed to the decoder layer through a combined-connection, to retrieve spatial information that has been lost due to downsampling. The left-leg input is concatenated to the right leg through the proposed long skip connections. In our proposed method, the long skip connections are first used to stabilize the gradient updates in the network. The right-leg layer accelerates convergence by reducing gradient vanishing problems and finally helps to produce more accurate segmentation results. The expression for the right leg is

(5) $$r^{\left[l\right]}=concatenate[upsample\left(r^{\left[l-1\right]}\right),d^{\left[l\right]},pool(x^{\left[l\right]},2]$$

where $r^{\left[l\right]}$ is the output of the right leg. All the feature maps obtained from the input of the last layer (left and bottom) before the softmax are concatenated into a combined feature map. The final network output feature maps are defined as

(6) $$f^{\left[l\right]}=concatenate\left[d^{\left[l\right]},\;r^{\left[l\right]}\right].$$

#### 3.2.3. Classification Layer

In the final decoder layer, a reconstructed segmentation map is predicted by the 1 × 1 convolutional layer with a softmax activation function. The output is classified into four categories: GM, WM, CSF, and background. The proposed network generates the corresponding learned representation from the input image. Using the feature representation, each input image is categorized into one of four output classes. We use the cross-entropy defined in (8) as a loss function. The softmax classifier which interprets the decoder representation into the output class, is used. The output class is given the probability score $f^{\left[l\right]}$. The number of output classes is defined as *c*, and then the predicted distribution score is obtained as (7).

(7) $$\hat{y}=\frac{exp^{f^{\left[l\right]}}}{\sum_{j=0}^{c}exp^{f_{j}^{\left[l\right]}}}$$

and the network cost function is computed using the cross-entropy loss function, which is represented as (8).

(8) $$L\left(y,\hat{y}\right)=-\sum_{i=0}^{c}y^{\left(i\right)}\;log\left({\hat{y}}^{\left(i\right)}\right)$$

where y and $\hat{y}$ are the ground truth and predicted distribution scores for each class I, respectively.

#### 3.2.4. Fire Module

The fire module is adopted in the proposed architecture in order to reduce the number of parameters for brain MRI segmentation; this module was initially developed to reduce the number of parameters for AlexNet [53] in order to maintain an acceptable classification accuracy. As part of the fire module of SqueezeNet [41], the squeeze layer is implemented using a 1 × 1 convolution filter to reduce the number of channels for the input elements. Further, an expanded layer utilizes 1 × 1 and 3 × 3 convolution filters to extract multi-scale information from the input image. In addition to features discussed in [41], we included a fire module as part of our proposed network based on three strategies. First, a 3 × 3 convolution filter is typically used in conventional networks [19,20,21]. In the proposed network, this is replaced by a squeeze layer that contains a 1 × 1 filter, which feeds into an expanding layer composed of a mixture of 1 × 1 and 3 × 3 convolution filters, as shown in Figure 3. As a result of using a 1 × 1 filter in the squeeze layer, there are nine times fewer parameters than those resulting from conventional 3 × 3 filters. The second reason is that fewer filters in the squeeze layer feeding into the expanded layer can reduce the total number of parameters in the network due to the reduced number of network connections. Finally, a number of conventional methods produce layers that have larger strides (>1), and most of the layers in the network have small activation maps. With this approach, we reduce the stride of the convolution layer to 1, making the feature maps in the network bigger and increasing segmentation accuracy.

#### 3.2.5. Training of OASIS and IBSR Datasets

The MRI scans and corresponding ground-truth labels for our proposed method were taken from publicly available datasets: open access series of imaging studies (OASIS) [54] and the internet brain segmentation repository (IBSR) [55]. In order to standardize the scan sizes (to 256 × 256 pixels), we divided the image into four uniform and non-overlapping patches by padding zeros across the boundaries of each slice. In the OASIS dataset, the axial scans had dimensions of 208 × 176 × 176 (size of each slice by height and width), and each axial scan was composed of 176 slices. In our experiment, the initial axial scan was resized to 256 × 256 × 176 dimensions by padding 24 pixels of zeros on the top and bottom and 40 pixels of zeros on the left and right of the image. There were several slices that contained no useful information at the beginning and end of the brain MRI volume, and almost identical data were exchanged between consecutive slices. Therefore, only 48 slices (each spaced by 3 slices) were extracted during the training process to remove repetitive slices and non-informative content. This resulted in input scans (and their corresponding ground-truth) having 256 × 256 × 48 dimensions. For training purposes, four uniform patches were created from each slice of an MRI and the ground truth associated with it. As a result, each divided patch in the proposed method has a size of 128 × 128. A segmented output was obtained using the test image when the partitioned patches were fed into the proposed model for training. A similar resizing was performed on the coronal (176 × 176× 208) and sagittal (176 × 208× 176) volumes of 256 × 256× 208 and 256 × 256× 176, respectively. In the IBSR dataset, all planes of brain MRI are resized to 256 × 256, and 48 slices with a three-slice gap are recovered from all planes of brain MRI.

## 4. Experimental Results and Analysis

### 4.1. Materials

The proposed method was verified using the brain MRI datasets such as OASIS [54] and IBSR [55]. Table 1 shows the details of these datasets.

#### 4.1.1. OASIS Dataset

The OASIS dataset [54] contains 416 demented and non-demented subjects with ages ranging from 18 to 96 years. For our experiment, the first 120 subjects were selected for training, while the last 30 were chosen randomly for testing.

#### 4.1.2. IBSR Dataset

The IBSR dataset [55] contains 18 high-resolution T1-weighted brain MRIs of 14 healthy males and 4 healthy females ranging in age from 7 to 71 years old. The skull stripping, normalization, and bias-field correction were used to pre-process the MRIs in the IBSR dataset. In the experiment, we chose the first 12 subjects for training and the remaining six subjects for testing. The subject volume of this dataset has dimensions of 256 × 256 × 128 and features distinct voxel spaces: 0.84 × 0.84 × 1.5 ${\mathrm{mm}}^{3}$, 0.94 × 0.94 × 1.5 ${\mathrm{mm}}^{3}$, and 1.0 × 1.0 × 1.5 ${\mathrm{mm}}^{3}$.

### 4.2. Experimental Setups

The proposed model was implemented using the Keras framework. The loss function was optimized using stochastic gradient descent on an NVIDIA GeForce RTX 3090 GPU for training and testing. A learning rate of 0.001, a high momentum rate of 0.99, a validation split of 0.2, and the number of 10 epochs for training were set, respectively. In the experiment, it was observed that the network loss function converged to its lowest value and appeared to overfit over 10 epochs. In addition, we stopped training a network when the validation error was as low as possible using early stopping [56]. Furthermore, the OASIS and IBSR datasets have an axial plane, with slices obtained from a transverse view. Using the ImageJ software [57], the slices were generated orthogonal to the axial plane with separate left and right sides to obtain sagittal plane images. To obtain coronal images, the slices were generated orthogonal to the sagittal plane. The following standard protocol was used to generate ground-truth images. The normalized whole-brain volume (nWBV) in the OASIS dataset was evaluated using the FAST program from the FMRIB software library (FSL) software package [58]. Initially, the image was segmented to differentiate between CSF, GM, and WM brain tissue. As part of the segmentation process, voxels were iteratively assigned to tissue types using a hidden Markov random field model that provides maximum likelihood estimates [59]. For each tissue class, a set of voxels of a given intensity were allocated based on spatial proximity. The number of voxels in the brain mask that were identified as GM or WM was computed as nWBV. The normalized volume is the proportion of all segmented voxels within an estimate of the total intracranial volume [60]. Similarly, the IBSR dataset provides expert-guided segmentation results, as well as MRI scans.

In order to objectively compare segmentation outputs to the ground truths, the Dice similarity coefficient (DSC) [61], the Jaccard index (JI) [62], and the Hausdorff distance (HD) [63] were used. The overlap between a given ground-truth map s and a predicted map $s^{\prime }$ was measured using the DSC and JI metrics, which are defined as

(9) $$DSC\left(s,s^{\prime }\right)=\frac{2\left|s\cap s^{\prime }\right|}{\left|s\left|+\right|s^{\prime }\right|},$$

(10) $$JI\left(s,s^{\prime }\right)=\frac{\left|s\cap s^{\prime }\right|}{\left|s\left|+\right|s^{\prime }\right|},$$

where the term ∩ refers to the overlap between the ground truth and the segmented map, while the |.| indicates the cardinality of the set. In addition, the maximum distances between points from one set and the nearest point from the other set using the Hausdorff distance (HD) were measured using (11).

(11) $$d\left(s,s^{\prime }\right)=max\left\{\underset{a\in s}{max}\underset{b\in s^{\prime }}{\;min}\left|b-a\right|,\underset{b\in s^{\prime }}{max}\underset{a\in s}{\;min}\left|a-b\right|\right\},$$

where a and b are the intersection of sets s and $s^{\prime }$, respectively. The HD between s and $s^{\prime }$ is the lowest number value d such that every point of s has a point of $s^{\prime }$ under *d* and vice versa.

### 4.3. Results for OASIS and IBSR Datasets

**Figure 4:**
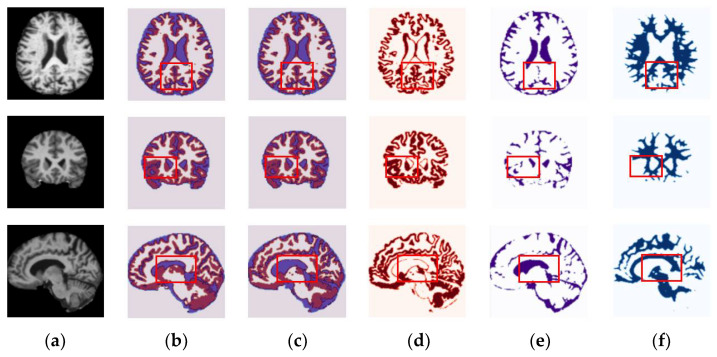
Randomly chosen test samples from brain MRI scans, showing segmentation results of the proposed method in all planes for the OASIS dataset: (**a**) original input images, (**b**) ground-truth maps, (**c**) predicted maps obtained under the proposed method, (**d**) predicted binary maps of GM, (**e**) predicted binary maps of CSF, and (**f**) predicted binary maps of WM.

**Figure 5:**
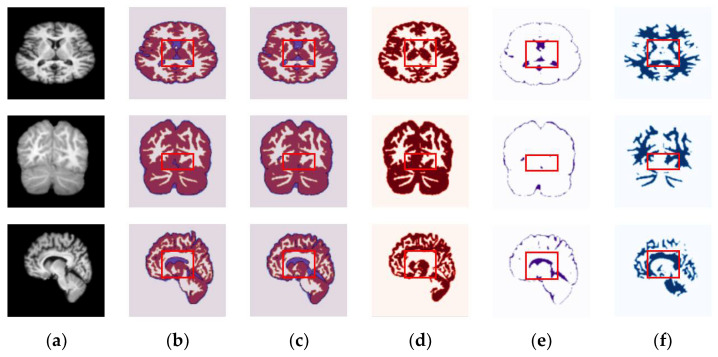
Randomly chosen test samples from brain MRI scans, showing segmentation results of the proposed method in all planes for the IBSR dataset: (**a**) original input images, (**b**) ground-truth map, (**c**) predicted maps obtained under the proposed method, (**d**) predicted binary maps of GM, (**e**) predicted binary maps of CSF, and (**f**) predicted binary maps of WM.

In order to verity the efficacy of the proposed method in different plane views, we present the segmentation results of the axial, coronal, and sagittal planes of the brain MRI. The orthogonal analysis of the brain with axial, coronal, and sagittal views is essential for a high-quality diagnosis. The biggest problem of the CNN networks for segmentation of the brain MRI lies in the number of images in the database. The MRI scans are acquired in different planes, so using all the available planes could enlarge the database. Further, we show that the proposed method is flexible to train and optimize on different image planes. Figure 4 and Figure 5 show segmentation results for the OASIS and IBSR datasets depicted for the axial, coronal, and sagittal planes, respectively. As shown in these figures, the proposed method achieves well-segmented accuracy for the GM, WM, and CSF of brain MRI in both datasets. The red rectangles in Figure 4 and Figure 5 are used to visualize the differences between segmentation results across the three planes obtained under the proposed method. They indicate that, particularly in the highlighted region, the axial plane produces a more accurate segmentation accuracy than the coronal and sagittal ones.

**Figure 6:**
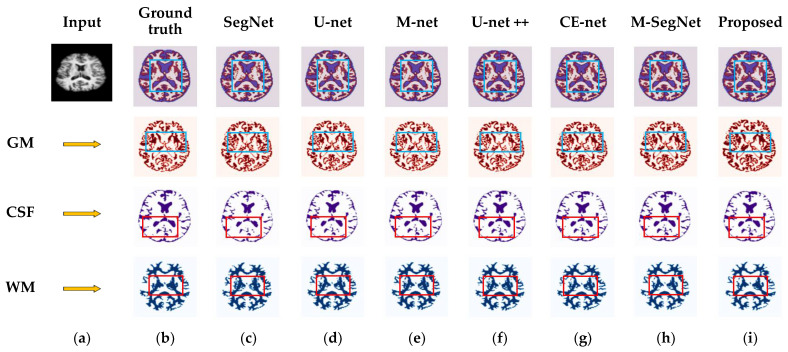
Qualitative comparison for GM, CSF, and WM using the proposed method and existing methods for the OASIS dataset. From left to right: (**a**) original input image; (**b**) ground-truth map; (**c**) segmentation results obtained by SegNet; (**d**) segmentation results obtained by U-net; (**e**) segmentation results obtained by M-net; (**f**) segmentation results obtained by U-net++; (**g**) segmentation results obtained by CE-Net; (**h**) segmentation results obtained by M-SegNet; (**i**) segmentation results obtained of the proposed SM-SegNet.

**Figure 7:**
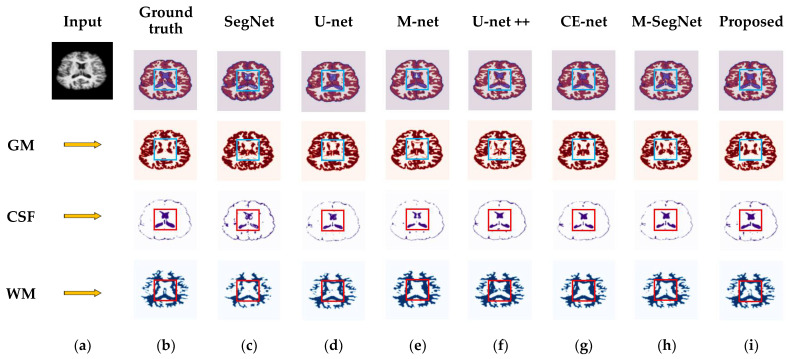
Qualitative comparison for GM, CSF, and WM using the proposed method and existing methods for the IBSR dataset. From left to right: (**a**) original input image; (**b**) ground-truth map; (**c**) segmentation results obtained by SegNet; (**d**) segmentation results obtained by U-net; (**e**) segmentation results obtained by M-net; (**f**) segmentation results obtained by U-net++; (**g**) segmentation results obtained by CE-Net; (**h**) segmentation results obtained by M-SegNet; (**i**) segmentation results obtained of the proposed SM-SegNet.

This is because, when compared to other planes in the MRI, the axial plane delivers the most informative features in the central slices. As a result, the network can be trained efficiently using the highest entropy values found in the central slices of the axial plane. Thus, using slices from the axial planes realizes the most effective segmentation performance. To demonstrate more specifically the segmentation effects of different network architectures, SegNet, U-net, M-net, U-net++, CE-Net, and M-SegNet models were trained on identical experimental data. Figure 6 and Figure 7 show the segmentation results for the various segmentation methods. As shown in Figure 6 and Figure 7, the quality of the proposed method’s segmentation results is markedly superior to those of the state-of-the-art methods. The segmentation results of SegNet and U-net models suffered greater spatial detail losses compared to the proposed method, as indicated by the red and blue squares in Figure 6c,d. As shown in Figure 6c, the SegNet performed an un-pooling operation from lower resolution feature maps. It lost adjacency information, resulting in a failure to capture fine details. Likewise, the U-net architecture was unable to capture detailed textures at tissue boundaries because low- and high-level features were mismatched during concatenation between decoding and encoding [64]. In U-net++, the network layers are connected by a series of nested dense skip paths, which leads to redundant feature learning. Hence, it did not perform well, as shown in Figure 6f. M-net features side paths that help to capture additional details unavailable to U-net; however, M-net still fails to preserve fine information across image boundaries. The CE-Net uses atrous convolution and associated multi-kernel max-pooling to collect data at multiple scales and prevent the redundant acquisition of data. On the other hand, the CE-Net can only extract multiple-scale features from the bottleneck layer, resulting in poor feature presentation in the final decoder layer. In M-SegNet, the network uses attention mechanisms and convolutional kernels of different sizes at the encoder and decoder stages and combined-connections to segment brain MRIs. Moreover, M-SegNet multi-scale deep networks assist with discriminative information extraction, and deep supervision facilitates model training by allowing for more learnable parameters.

The multi-scale information extracted at the bottleneck layer may be irrelevant over large distances. Meanwhile, because the proposed method extracts discriminative information through multi-scale side paths, the combined-connections are used to restore the spatial information, the long skip connections are used to stabilize the gradient updates in the network, and the uniform input patches allow the network to preserve fine local details; this can realize superior segmentation accuracies compared to existing methods, which use whole slices as inputs. The experiment was also performed on 18 T1-weighted brain MRI scans from the IBSR dataset. In this dataset, the original ground-truth annotation does not contain sulcal parts of the CSF tissue, in contrast to the GM [65]. These were used to evaluate the segmentation accuracy [66]. In our study, we performed experiments for all methods using the original IBSR dataset without additional annotations. As a result, the mean DSC values of the CSF exhibited low segmentation performances in comparison to the OASIS dataset results. Figure 7 shows the segmented results of the CSF, GM, and WM delineations obtained by the conventional and proposed methods. As highlighted by the red squares, the anatomical details captured by the proposed method are more consistent with the ground truth than those observed using other recent methods. Table 2 compares the segmentation results between the conventional and proposed methods for the OASIS and IBSR datasets. As measured by the DSC, JI, and HD metrics, the proposed method exhibits a significantly higher segmentation accuracy than existing methods. Our proposed network achieved a mean improvement of 7%, 4%, 3%, 2%, 1.5%, and 0.5% (in terms of DSC) with respect to SegNet [19], U-net [20], M-net [21], U-net++ [34], CE-Net [36], and M-SegNet [39] methods, respectively. This is because the proposed method uses information extracted from multi-scale side paths. Additionally, the combined-connections can preserve the spatial information of the feature maps during downsampling. Hence, combined-connections and long skip connections use the stored information to upsample the feature maps and automatically capture complex structural patterns. In addition, the input slices are divided into patches to preserve the fine local details; this leads to a superior segmentation accuracy compared to existing methods, which use whole slices as inputs. The fire module adopted in our method significantly reduces the number of parameters.

**Figure 8:**
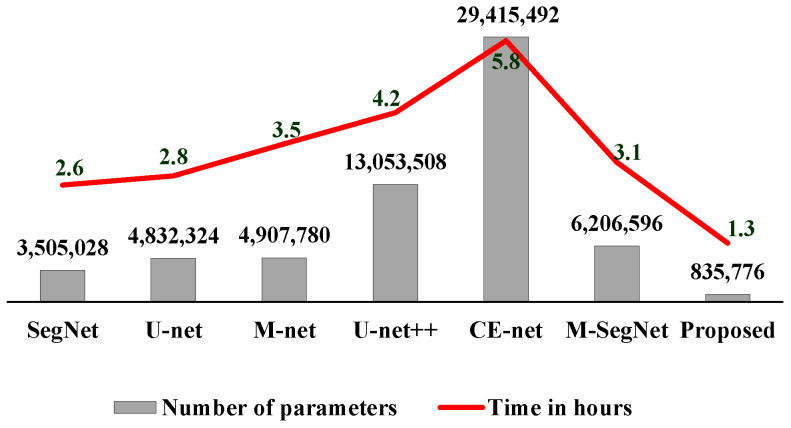
Comparison of the proposed and conventional methods in terms of the number of trainable parameters and computation times.

Figure 8 shows the number of learnable parameters and the computational complexity required by the proposed method in comparison to existing methods. In Figure 8, the computation time is the total time required to construct the training model under the experimental conditions. Our proposed method required 835,776 parameters (i.e., below 1 × 10^6^), 4 times fewer than SegNet, 6 times fewer than U-net and M-net, 15 times fewer than U-net++, 7 times fewer than M-SegNet, and 35 times fewer than CE-Net. A training time of 50% was obtained for the proposed method for the OASIS dataset, as compared with U-net++ and M-net. Owing to the use of the fire module in the proposed method, large reduction in the number of learnable parameters while retaining good segmentation accuracy. In terms of computation time, our proposed method takes ~1.3 h to train the proposed model and ~2 min to test each input slice.

### 4.4. Ablation Study

As shown in Figure 3, the proposed model was applied to axial, coronal, and sagittal slices for brain MRI segmentation. We conducted an ablation study on the OASIS dataset to demonstrate the contribution of the proposed network. To investigate the effectiveness of our proposed model, we performed experiments on four simplified brain-MRI-segmentation models: (1) M-SegNet architecture only, (2) SM-SegNet without long skip connections, (3) M-SegNet with long skip connections, and (4) SM-SegNet with long skip connections (all-combined proposed method). Table 3 presents the segmentation accuracy in terms of the DSC and JI metrics for the proposed simplified segmentation models. The multi-scale information passed from the left leg to the right leg through long skip connections, which improved the reconstruction of the output. Hence, the M-SegNet model using long skip connections exhibited a 2% increased accuracy compared to the case without long skips. Alongside accuracy, computational complexity is also an important metric for the evaluation of segmentation tasks. From the results in Table 3, the SM-SegNet implemented with the fire module can be seen to reduce the number of learnable parameters, reducing the computation time and memory requirements. The SM-SegNet requires 835,776 learnable parameters, 7 times fewer than the M-SegNet architecture. The proposed method was also observed to attain the highest accuracy compared to other simplified models, and it required the smallest number of parameters owing to its efficient memory usage; therefore, it is faster to train than the other models.

To evaluate the segmentation performance and model training time, Table 4 shows the effects of different input-patch sizes. Experiments were performed on the OASIS dataset for three different patch sizes (i.e., 32 × 32, 64 ×64, and 128 × 128). It can be observed that, compared to the other patch sizes (32 × 32, 64 × 64), the patch size of 128 × 128 in the proposed method requires a smaller training time (1 h). The DSC values for the smaller patch size of 32 × 32 are found to achieve superior performance because the proposed architecture can be trained on 16 times more data with 32× 32 patches than 128 × 128 patches. Thus, smaller patch sizes (32 × 32 and 64 × 64) can improve the segmentation accuracy of the brain MRI while it requires more computational time than those of 128 × 128 patches. The experimental results indicate that the difference in segmentation accuracy between the smaller and larger patch sizes is marginal. As a result, we empirically chose to use a patch size of 128 × 128 to train our model to compromise the segmentation accuracy and computation efficiency. Therefore, a 128 × 128 patch size was determined as a good choice, representing a fair tradeoff between the DSC values compared with smaller patch sizes. The experiments were conducted, as shown in Table 5, to investigate the effects of non-overlapping patches on overlapping patches for brain MRI segmentation under the proposed method. In our experimental analysis, an input size of 128 × 128 was used for both non-overlapping and overlapping patches in brain MRI segmentation. For the overlapping patch process, an optimal stride of 8 pixels was selected; this was because a pixel stride of less than 8 pixels produces identical segmentation results because similar information is shared by overlapping patches with a small stride difference. Furthermore, a smaller pixel stride also results in more patches, which increases computational complexity as well.

As shown in Table 5, although the overlapping method segmented with nearly the same accuracy as the non-overlapping method (with a DSC value of 0.97 and JI value of 0.94), the predicted output was not accurately reconstructed. As a result, multiple convolution operations are performed over the same element of the pixel. Since each overlapping patch must be trained separately, the overlapping patches approach requires more computation time. In our experimental setup, the overlapping method requires 28.5 h of training, whereas the proposed method requires only 1.3 h.

To compare the proposed method with conventional methods, we selected the state-of-the-art methods recently presented, which use the same datasets as our proposed method. Table 6 shows the comparison of our proposed method to state-of-the-art methods on publicly available datasets (IBSR and OASIS). Bao et al. [67] introduced a new technique for segmenting brain images based on multi-scale CNN, which provides differentiated features for a given subcortical structure, and generates a probability map to label the target image. As a result, there would be no spatial constraints in the testing samples since the brain images have an irregular background. Khagi et al. [68] used a SegNet architecture that is based on CNN to segment cross-sectional brain images. With the simplified SegNet architecture approach, pixels with heterogeneously distributed class labels are segmented based on their labels. Shakeri et al. [69] introduced the semantic segmentation of objects in natural images using FCNN architecture and show improved results by interpreting CNN output as possibilities of a Markov random field, whose topology corresponds to a volumetric grid. Dolz et al. [70] used the 3D CNN architecture to segment subcortical MRI brain structures and handled computational complexity and memory requirements well. The results indicate that the proposed method is significantly more accurate than the previous methods in terms of segmentation accuracy. It is noted that the results in Table 6 show comparisons based on the entire algorithms, while the results in Table 2 show the comparisons based on the different deep learning architectures. There are several methods [71,72,73,74,75] using the Clinical and BrainWeb datasets [76]. These CNN methods [71,72,73,74,75] provide segmentation accuracy from 0.85 to 0.94. Due to the difference in experimental conditions, the segmentation results details are not included in this paper.

The 3D segmentation method generates a dense segmentation from the annotations of specific slices in the 3D volume. However, brain segmentation in 3D space requires more complex analysis than in 2D space, which is the reason that it is less adaptable to user interactions [77]. Moreover, it is also stated in [78] that interactive 2D segmentation is more appropriate than direct 3D segmentation due to the huge interslice spacing and motion of the images. In comparison with manipulating patches in 2D, our proposed method is significantly more computationally complicated for 3D patches. The parallel segmentation of thousands of 3D volumetric images requires large computational complexity due to the limited number of parallel processing nodes and sub-processes. Furthermore, our proposed network produces significantly fewer parameters with efficient memory utilization, higher inference speed, and greater transferability of information for brain MRI segmentation. Based on the aforementioned reasons, to compensate for the lack of contextual information in 2D space, we propose the 2D network inputs by feeding uniform patches from the slices into 2D networks.

To investigate the impact of the cross-validation scheme, we performed the experiment on the IBSR dataset. A training set of 12 subjects and a test set of 6 subjects were constructed for the experiment by using the random partition with a particular split ratio. Table 7 shows the summary of the segmentation results of brain MRI using the cross-validation approach for IBSR datasets. As shown in Table 7, we can observe that the model trained on TestSet0 with the last 12 subjects and tested on the first 6 subjects shows an average DSC score of 0.86 and JI score of 0.75 for segmentation of brain MRI. Similarly, the model trained on TestSet1 with the first half and second half of the datasets shows an average DSC score of 0.87 and JI score of 0.77, and the model trained on TestSet2 with the first 12 subjects shows an average DSC score of 0.88 and JI score of 0.79 for segmentation of brain tissues. This demonstrates that our proposed method was consistent in terms of DSC and JI evaluation metrics for the segmentation of brain MRI regardless of the types of datasets.

Further, the Wilcoxon rank-sum test was used to assess whether the proposed method is significantly better than conventional methods. In Table 8, the *p*-values indicate that our proposed method achieves a significant improvement over conventional methods at the 5% level (all *p*-values were less than 0.05).

**Figure 9:**
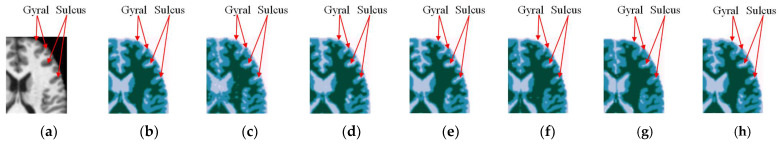
Simplified inner cortical surfaces illustrating gyral and sulcus regions of the brain MRI. (**a**) Original input images, (**b**) ground-truth segmentation maps, (**c**) predicted segmentation map of SegNet, (**d**) predicted segmentation map of U-net, (**e**) predicted segmentation map of M-net, (**f**) predicted segmentation map of U-net++, (**g**) predicted segmentation map of CE-net and (**h**) predicted segmentation map of the proposed method.

To investigate the inner cortical surface regions from different methods and ground truth, we show the gyral and sulcus regions in Figure 9. The gyral and sulcus regions of the brain MRI assists in defining the location of brain function on the cortex, which can be used to learn more about brain function or to avoid critical areas in neurosurgery. In Figure 9, we observe that the proposed method has fewer anatomical errors compared to existing methods.

**Figure 10:**
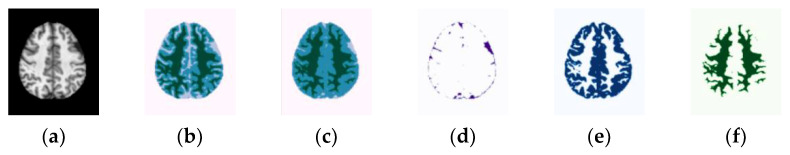
Segmentation results of the proposed method trained for the IBSR dataset and tested on OASIS data: (**a**) original input images, (**b**) ground-truth segmentation maps, (**c**) predicted segmentation maps obtained under the proposed method, (**d**) predicted binary maps of CSF, (**e**) predicted binary maps of GM, and (**f**) predicted binary maps of WM.

**Figure 11:**
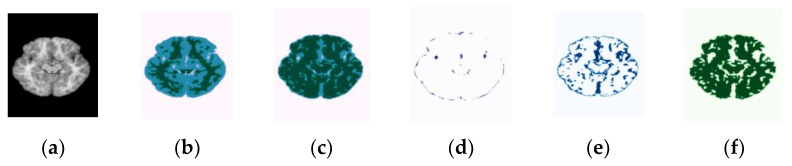
Segmentation results of the proposed method trained for the OASIS dataset and tested on IBSR data: (**a**) original input images, (**b**) ground-truth segmentation maps, (**c**) predicted segmentation maps obtained under the proposed method, (**d**) predicted binary maps of CSF, (**e**) predicted binary maps of GM, and (**f**) predicted binary maps of WM.

To evaluate the effectiveness of the proposed method, we conducted the experiments by applying a model trained on one dataset to the other dataset. As shown in Table 9, we can observe that the model trained on IBSR with the 18 subjects and tested on the 15 subjects shows an average DSC score of 0.77 for segmentation of brain tissues on MRI. In a similar way, the model trained on the OASIS dataset with 50 subjects and tested on the IBSR dataset with 18 subjects shows an average DSC score of 0.60. Figure 10 and Figure 11 show the segmentation results of the proposed method.

The prediction obtained for IBSR test data shows a low DSC score for brain tissue segmentation because of poor quality images in the IBSR dataset compared to the OASIS dataset. Further, unlike the results on the OASIS test dataset, the mean DSC scores for CSF show low values on the IBSR test dataset because original ground-truth annotations in the IBSR do not contain sulcal parts of CSF tissue, unlike GM.

## 5. Conclusions

In this paper, we proposed a new SM-SegNet architecture that can realize an improved performance compared to conventional methods for brain MRI segmentation. High segmentation accuracy can be achieved and fine local details can be preserved using uniformly partitioned input patches. In the proposed architecture, the multi-scale side input layer helps to extract discriminative information, and deep supervision on the output side and leads to faster convergence of the model through long skip connections. Furthermore, the use of fire modules in the encoder and decoder paths of the proposed architecture reduces the learnable parameters and realizes a memory-efficient segmentation model. Compared to conventional approaches, the reduced memory requirements of our model substantially lower the computational processing requirements. Finally, our results show that the proposed approach can reach average DSC and JI values of 0.96 and 0.92, respectively, which is an improvement over existing methods.

## Figures and Tables

**Table 1:** Details of OASIS and IBSR datasets.

Categories	Number of Subjects	
	OASIS	IBSR
Males	160	14
Females	256	4
Total	**416**	**18**

**Table 2:** Segmentation accuracy comparison for the proposed and conventional methods on OASIS and IBSR datasets.

OASIS									
Axial Plane									
Methods	WM			GM			CSF		
	DSC	JI	HD	DSC	JI	HD	DSC	JI	HD
SegNet [19]	0.89 ± 0.087	0.80 ± 0.096	4.74 ± 0.077	0.86 ± 0.069	0.75 ± 0.089	4.69 ± 0.053	0.85 ± 0.048	0.74 ± 0.068	4.12 ± 0.079
U-net [20]	0.93 ± 0.059	0.87 ± 0.068	4.16 ± 0.064	0.92 ± 0.048	0.85 ± 0.061	4.24 ± 0.046	0.90 ± 0.076	0.82 ± 0.090	3.82 ± 0.039
M-net [21]	0.94 ± 0.046	0.89 ± 0.057	4.02 ± 0.023	0.93 ± 0.055	0.87 ± 0.072	4.11 ± 0.077	0.92 ± 0.044	0.85 ± 0.065	3.79 ± 0.043
U-net++ [34]	0.95 ± 0.053	0.90 ± 0.062	3.78 ± 0.048	0.94 ± 0.035	0.89 ± 0.048	3.84 ± 0.025	0.93 ± 0.039	0.87 ± 0.052	3.56 ± 0.036
CE-Net [36]	0.95 ± 0.039	0.90 ± 0.044	3.65 ± 0.050	0.95 ± 0.042	0.90 ± 0.057	3.57 ± 0.044	0.93 ± 0.043	0.87 ± 0.063	3.21 ± 0.061
M-SegNet [39]	0.96 ± 0.030	0.92 ± 0.053	3.28 ± 0.041	0.96 ± 0.033	0.92 ± 0.048	3.25 ± 0.026	0.95 ± 0.029	0.90 ± 0.042	3.08 ± 0.032
**Proposed**	0.97 ± **0.032**	0.94 ± **0.040**	3.31 ± **0.028**	0.96 ± **0.027**	0.92 ± **0.034**	3.88 ± **0.019**	0.95 ± **0.021**	0.90 ± **0.036**	2.83 ± **0.015**
**Coronal Plane**									
SegNet [19]	0.87 ± 0.058	0.77 ± 0.065	5.21 ± 0.023	0.85 ± 0.044	0.74 ± 0.068	5.49 ± 0.053	0.83 ± 0.056	0.71 ± 0.074	5.87 ± 0.084
U-net [20]	0.94 ± 0.043	0.89 ± 0.059	4.88 ± 0.042	0.93 ± 0.057	0.87 ± 0.069	4.95 ± 0.042	0.92 ± 0.063	0.85 ± 0.081	5.34 ± 0.073
M-net [21]	0.94 ± 0.048	0.89 ± 0.060	4.33 ± 0.066	0.92 ± 0.021	0.85 ± 0.032	4.33 ± 0.038	0.92 ± 0.034	0.85 ± 0.051	4.90 ± 0.032
U-net++ [34]	0.94 ± 0.066	0.89 ± 0.073	4.05 ± 0.047	0.93 ± 0.042	0.87 ± 0.057	4.29 ± 0.044	0.93 ± 0.048	0.87 ± 0.059	4.72 ± 0.043
CE-Net [36]	0.95 ± 0.031	0.90 ± 0.046	3.98 ± 0.076	0.94 ± 0.038	0.89 ± 0.050	4.17 ± 0.071	0.93 ± 0.039	0.87 ± 0.053	4.17 ± 0.050
M-SegNet [39]	0.96 ± 0.024	0.92 ± 0.038	3.43 ± 0.046	0.95 ± 0.024	0.90 ± 0.036	3.48 ± 0.066	0.94 ± 0.032	0.89 ± 0.048	3.64 ± 0.036
**Proposed**	0.96 ± **0.023**	0.92 ± **0.032**	3.22 ± **0.038**	0.95 ± **0.033**	0.90 ± **0.046**	3.29 ± **0.054**	0.94 ± **0.044**	0.89 ± **0.062**	3.37 ± **0.028**
**Sagittal plane**									
SegNet [19]	0.88 ± 0.054	0.79 ± 0.066	5.53 ± 0.027	0.85 ± 0.083	0.74 ± 0.095	5.26 ± 0.033	0.84 ± 0.040	0.72 ± 0.057	5.69 ± 0.088
U-net [20]	0.94 ± 0.058	0.89 ± 0.070	5.11 ± 0.030	0.92 ± 0.074	0.85 ± 0.090	5.11 ± 0.026	0.93 ± 0.058	0.87 ± 0.073	5.21 ± 0.079
M-net [21]	0.94 ± 0.038	0.89 ± 0.045	5.34 ± 0.046	0.92 ± 0.083	0.85 ± 0.094	4.67 ± 0.026	0.93 ± 0.029	0.87 ± 0.046	5.04 ± 0.082
U-net++ [34]	0.95 ± 0.060	0.90 ± 0.072	4.46 ± 0.031	0.94 ± 0.038	0.89 ± 0.049	4.32 ± 0.019	0.94 ± 0.041	0.89 ± 0.063	4.56 ± 0.041
CE-Net [36]	0.95 ± 0.043	0.90 ± 0.064	4.13 ± 0.020	0.94 ± 0.025	0.89 ± 0.037	4.25 ±0.034	0.94 ± 0.051	0.89 ± 0.062	4.28 ± 0.055
M-SegNet [39]	0.95 ± 0.029	0.90 ± 0.047	3.68 ± 0.035	0.95 ± 0.021	0.90 ± 0.035	3.16 ± 0.042	0.95 ± 0.036	0.90 ± 0.047	3.79 ± 0.027
**Proposed**	0.95 ± **0.035**	0.90 ± **0.044**	3.46 ± **0.038**	0.95 ± **0.044**	0.90 ± **0.056**	3.09 ± **0.035**	0.95 ± **0.058**	0.90 ± **0.073**	3.46 ± **0.045**
**Axial Plane**									
SegNet [19]	0.72 ± 0.036	0.56 ± 0.042	6.51 ± 0.65	0.75 ± 0.049	0.60 ± 0.058	6.53 ± 0.91	0.78 ± 0.079	0.64 ± 0.095	6.96 ± 0.46
U-net [20]	0.89 ± 0.022	0.80 ± 0.034	5.14 ±0.51	0.91 ± 0.027	0.83 ± 0.038	4.87 ± 0.51	0.84 ± 0.062	0.72 ± 0.079	5.24 ± 0.31
M-net [21]	0.90 ± 0.043	0.82 ± 0.051	4.76 ± 0.39	0.92 ± 0.053	0.85 ± 0.068	4.45 ± 0.65	0.84 ± 0.039	0.72 ± 0.048	4.84 ± 0.18
U-net++ [34]	0.88 ± 0.085	0.79 ± 0.096	5.37 ± 0.36	0.89 ± 0.037	0.80 ± 0.049	5.17 ± 0.29	0.83 ± 0.058	0.71 ± 0.072	5.34 ± 0.64
CE-Net [36]	0.89 ± 0.055	0.80 ± 0.073	4.98 ± 0.84	0.90 ± 0.068	0.82 ± 0.083	4.95 ± 0.38	0.82 ± 0.037	0.69 ± 0.054	4.74 ± 0.93
M-SegNet [39]	0.90 ± 0.038	0.82 ± 0.049	4.59 ± 0.64	0.92 ± 0.055	0.85 ± 0.028	4.43 ± 0.47	0.84 ± 0.032	0.72 ± 0.055	4.42 ± 0.24
**Proposed**	0.91 ± **0.042**	0.83 ± **0.054**	4.45 ± **0.57**	0.93 ± **0.026**	0.87 ± **0.040**	4.23 ± **0.92**	0.85 ± **0.026**	0.74 ± **0.039**	4.26 ± **0.79**
**Coronal Plane**									
SegNet [19]	0.70 ± 0.043	0.54 ± 0.052	6.32 ± 0.82	0.73 ± 0.037	0.57 ± 0.052	6.21 ± 0.84	0.76 ± 0.064	0.61 ± 0.086	6.84 ± 0.75
U-net [20]	0.88 ± 0.035	0.79 ± 0.046	5.45 ± 0.67	0.90 ± 0.044	0.82 ± 0.056	5.17 ± 0.38	0.83 ± 0.028	0.71 ± 0.043	5.54 ± 0.47
M-net [21]	0.89 ± 0.046	0.80 ± 0.058	4.61 ± 0.21	0.91 ± 0.035	0.83 ± 0.043	4.56 ± 0.19	0.84 ± 0.075	0.72 ± 0.093	4.83 ± 0.25
U-net++ [34]	0.88 ± 0.059	0.79 ± 0.073	5.21 ± 0.39	0.91 ± 0.063	0.83 ± 0.078	5.24 ± 0.24	0.82 ± 0.048	0.69 ± 0.067	5.73 ± 0.39
CE-Net [36]	0.89 ± 0.054	0.80 ± 0.066	4.89 ± 0.21	0.90 ± 0.049	0.82 ± 0.068	5.98 ± 0.93	0.83 ± 0.056	0.71 ± 0.072	5.21 ± 0.20
M-SegNet [39]	0.91 ± 0.026	0.83 ± 0.043	4.39 ± 0.42	0.92 ± 0.071	0.85 ± 0.040	4.52 ± 0.36	0.83 ± 0.033	0.71 ± 0.047	4.26 ± 0.52
**Proposed**	0.90 ± **0.039**	0.82 ± **0.051**	4.24 ± **0.43**	0.92 ± **0.019**	0.85 ± **0.032**	4.31 ± **0.67**	0.84 ± **0.022**	0.72 ± **0.034**	4.55 ± **0.12**
**Sagittal Plane**									
SegNet [19]	0.71 ± 0.036	0.55 ± 0.048	6.49 ± 0.61	0.74 ± 0.073	0.59 ± 0.089	6.36 ± 0.76	0.75 ± 0.073	0.60 ± 0.092	6.99 ± 0.41
U-net [20]	0.86 ± 0.049	0.75 ± 0.062	5.75 ± 0.37	0.89 ± 0.036	0.80 ± 0.045	5.77 ± 0.21	0.80 ± 0.071	0.67 ± 0.089	5.83 ± 0.15
M-net [21]	0.87 ± 0.026	0.77 ± 0.038	4.89 ± 0.14	0.90 ± 0.045	0.82 ± 0.062	5.42 ± 0.06	0.81 ± 0.056	0.68 ± 0.073	4.98 ± 0.09
U-net++ [34]	0.85 ± 0.033	0.74 ± 0.045	4.57 ± 0.54	0.88 ± 0.063	0.79 ± 0.081	4.96 ± 0.22	0.79 ± 0.049	0.65 ± 0.070	5.60 ± 0.44
CE-Net [36]	0.86 ± 0.054	0.75 ± 0.065	5.34 ± 0.66	0.89 ± 0.051	0.80 ± 0.077	5.86 ± 0.55	0.79 ± 0.033	0.65 ± 0.045	5.25 ± 0.37
M-SegNet [39]	0.89 ± 0.032	0.80 ± 0.049	4.46 ± 0.52	0.90 ± 0.029	0.82 ± 0.042	5.42 ± 0.31	0.82 ± 0.020	0.69 ± 0.035	4.31 ± 0.32
**Proposed**	0.88 ± **0.035**	0.79 ± **0.053**	4.63 ± **0.36**	0.91 ± **0.028**	0.83 ± **0.043**	5.30 ± **0.18**	0.82 ± **0.024**	0.69 ± **0.039**	4.12 ± **0.66**

**Table 3:** Performance comparison between the simplified proposed models using axial plane for the OASIS dataset.

Methods	WM		GM		CSF		Parameters	Training Time
	DSC	JI	DSC	JI	DSC	JI		
**M-SegNet only**	0.94	0.89	0.95	0.90	0.94	0.89	5,468,932	3.09 h
**SM-SegNet without long skip**	0.95	0.90	0.94	0.89	0.94	0.89	**835,770**	1.50 h
**M-SegNet with long skip**	0.96	0.92	0.95	0.90	0.94	0.89	5,468,944	3.15 h
**Combined**	**0.97**	**0.94**	**0.96**	**0.92**	**0.95**	**0.90**	**835,776**	**1.30 h**

**Table 4:** Segmentation accuracy and training time (hours) for the proposed method under different input-patch sizes using the axial plane on the OASIS dataset.

Patch Size	WM			GM			CSF			Training Time
	DSC	JI	HD	DSC	JI	HD	DSC	JI	HD	
**32** × **32**	0.98	0.96	3.10	0.97	0.94	3.05	0.96	0.92	3.15	13.90 h
**64** × **64**	0.98	0.96	3.16	0.97	0.94	3.09	0.95	0.90	3.19	6.50 h
**128** × **128**	**0.97**	**0.94**	**3.25**	**0.96**	**0.92**	**3.15**	**0.95**	**0.90**	**3.22**	**1.30 h**

**Table 5:** Effects of overlapping and non-overlapping patch-wise segmentation in the proposed method.

No.	Parameters	Overlapping Patches	Non-Overlapping Patches
1	Input size	128 × 128	128 ×128
2	Training set	120 subjects	120 subjects
3	Testing set	30 subjects	30 subjects
4	# of patches	32 (stride: 8 pixels)	4
5	# of epochs	10	10
6	DSC	0.97	0.96
7	JI	0.94	0.92
8	Training time	28.5 h	1.3 h

**Table 6:** DSC and JI scores achieved by the proposed method in comparison with the state-of-the-art methods based on the publicly available database.

Methods		DSC and JI			Datasets	Features
		GM	WM	CSF		
1	Bao [67]	0.85	0.82	0.82	IBSR	Multi-scale structured CNN
2	Khagi [68]	0.74	0.81	0.72	OASIS	Simplified SegNet architecture
3	Shakeri [69]	0.82	0.82	0.82	IBSR	Multi-label segmentation using fully CNN (FCNN)
4	Dolz [70]	0.90	0.90	0.90	IBSR	3D FCNN
5	Proposed	0.96	0.97	0.95	OASIS	Patch-wise-based SM-SegNet architecture
		0.92	0.90	0.83	IBSR	

**Table 7:** Segmentation results obtained using the cross-validation approach, which randomly selects MRIs of the IBSR datasets.

Sets	Training (Subject #)	Test (Subject #)	Parameter	GM	WM	CSF
TestSet0	6–17	0–5	DSC	0.91	0.88	0.79
			JI	0.83	0.79	0.65
TestSet1	0–5 and 12–7	6–11	DSC	0.90	0.90	0.80
			JI	0.82	0.82	0.67
TestSet2	0–11	12–17	DSC	0.92	0.89	0.83
			JI	0.85	0.80	0.71

**Table 8:** *p*-values measured using Wilcoxon rank-sum analysis on the OASIS dataset.

**Metrics**	**SegNet** **vs.** **Proposed**	**U-Net** **vs.** **Proposed**	**M-Net** **vs.** **Proposed**	**U-Net++** **vs.** **Proposed**	**CE-Net** **vs.** **Proposed**
DSC	0.018	0.026	0.029	0.034	0.038

**Table 9:** Segmentation performance for brain MRI applying the proposed model trained on one dataset to the other dataset.

Model	Training Set	Test Set	DSC		
			GM	WM	CSF
Proposed	IBSR—18 Subjects	OASIS—15 Subjects	0.81	0.88	0.63
	OASIS—50 Subjects	IBSR—18 Subjects	0.60	0.67	0.54

## Data Availability

Not applicable.
